# A review on tailoring metal–organic frameworks for electrochemical applications: Advances in synthesis and structure for energy storage

**DOI:** 10.1016/j.isci.2025.113627

**Published:** 2025-09-22

**Authors:** Veerakumari P., Venkateswara Rao A., Anjani Devi K., Venkata Rao P., Raziya Shaik, Shanmugan S., Arunkumar Jayakumar, Karthik Kannan

**Affiliations:** 1 Department of Physics, Advanced Functional Materials Research Centre, Koneru Lakshmaiah Education Foundation, Guntur, Andhra Pradesh 522302, India; 2 Department of Physics, Aditya University, Surampalem, Andhra Pradesh 533437, India; 3 Department of Chemical Engineering, AU College of Engineering, Visakhapatnam, Andhra Pradesh 530003, India; 4 Department of Chemistry (Bs&H), Visakha Institute of Engineering & Technology (A) Narava, Visakhapatnam, Andhra Pradesh 530027, India; 5 Research Centre for Solar Energy, Department of Integrated Research & Discovery-Physics, Koneru Lakshmaiah Education Foundation, Vaddeswaram, Andhra Pradesh, India; 6 St.Peter’s Institute of Higher Education and Research, Avadi, Chennai, Tamil Nadu 600054, India; 7 Institute of Agricultural Engineering, Saveetha School of Engineering, Saveetha Institute of Medical and Technical Sciences, Chennai, Tamil Nadu 602105, India

**Keywords:** Applied sciences, Energy storage, Materials synthesis

## Abstract

Ions of metal and organic ligands combine to create the captivating family of metal-organic frameworks (MOFs), which are crystallized materials with pores. MOFs have potential applications in supercapacitors due to their appealing qualities. For supercapacitor and water splitting applications, this review aims to determine the elements influencing MOF features and their impact on structural and electrochemical characteristics. Supercapacitors, referred to as SCs, are regarded as exceptional energy-conserving devices due to high power density (Pd) and long cycling life. The key process in SCs is energy storage, which ensures rate ability and extended cycling through efficient operation. Many reviews focus on electrolytes, electrode materials, and SC applications. A detailed report on MOFs as electrode materials for SCs is included in this review. The main goal is to present a concise overview of recent advancements in the field of pure MOFs and related forms, including MOF derivatives and MOF composites.

## Introduction

Metal-organic frameworks (MOFs) are a fascinating and rapidly expanding class of crystalline, porous materials that have garnered significant attention in various scientific and technological fields. Often referred to as “wonder materials” of the 21st century, MOFs exhibit a unique combination of properties that make them highly versatile. At their core, MOFs are hybrid organic-inorganic materials constructed from two main components: Metal ions or metal clusters (nodes): These act as the inorganic “joints” or “building blocks” of the framework. They can be various transition metals, alkaline earth metals, or even lanthanides. Organic ligands (linkers): These are multi-directional organic molecules that bridge the metal nodes, forming an extended, repeating network. Common organic linkers include carboxylates, phosphonates, sulfonates, and various heterocyclic compounds. The literature study shows that different metal nodes showed different properties in applications.

MOFs based on cobalt (Co) are porous materials that have garnered a lot of interest in the field of electrocatalysis because of their huge specific surface areas, changeable pores, and programmable structure. Co-based MOFs for HER over the last several years, compared to noble metals, cobalt is inexpensive and abundant. Co-based composites are great candidates for HER because of their low energy barrier for H adsorption, which also contributes to their high theoretical catalytic activity. Consequently, a lot of research has been done on Co-based catalysts as HER catalysts. Co-based MOFs have garnered a lot of interest because they can produce extremely effective HER catalysts.^1^

Qiang Xu et al. created superlong Co-MOF nanotubes with individual crystals that have an overall length of about 20–35 μm and an average diameter of about 70 nm with a corresponding multi-channel (Size of the window: 1.1 nm). The resultant stacked dendrites to the branches of carbon nanotubes of carbon and nanofibers possess outstanding activity of electrocatalysis for the reduction of oxygen Reactions and remarkable uses in rechargeable Zn-air batteries.^2^

MOFs play a key role in electrochemical applications because of their large surface area, redox-active metal centers, chemical tunability, and adjustable porosity. These characteristics improve electrode–electrolyte interfaces, promote effective ion transport, and enable precise functionalization for certain electrochemical procedures such as electrocatalysis, batteries, fuel cells, and supercapacitors. Furthermore, MOFs can be designed to have structural stability and electronic conductivity, both of which are necessary for energy devices to cycle over an extended period.^3^

Lucio Angnes et al.^4^ reported that MOFs are being extensively reported as ideal templates or precursors for energy storage and conversion materials thanks to their unique architectures with high surface area, high ordered porosity, the concentration of heteroatoms, and adjustable structures, allied with the possibility of carrying out chemical processes while preserving their structure and enhancing or incorporating specific properties, and essential features for the rational design of multifunctional electrode materials for energy technologies.

MOF-5 crystals were produced by Zhu et al.^5^ on Cellulose-Based Substrates, such as cotton and paper. The TiO_2_@MOF Core–Shell Nanorod set is created by applying an 8 nm thickness MOF layer using the layered-by-layer self-assemble technique to a vertically arranged TiO_2_ nanorod set Scaffold, as described by Nianqiang Wu et al.^6^ In order to extract (Holes)Minority charge carriers produced by photolysis in TiO_2_ and transfer them regarding electrolytes, this set of vertically oriented Core-Shell Nanorods allows for a long wavelength of the small route length yet a visual path. When exposed to a 300 W Xe light without filtering that has a 100 mW/cm^2^ density of output power, the average Photocurrent Density of a TiO_2_@Co-Metal Organic Frameworks Nanorod in the Photo Electrochemical Cell set Anode photoelectric is 2.93 mA/cm^2^ at 1.23 V in relation to RHE, which is approximately 2.7 multiples the photo current obtained in a naked TiO_2_ set of nanorods of data. The reticular synthesis produces MOFs, whose precise ingredient selection can result in excellent thermal and Chemical Durability along with Crystals of Ultrahigh Porosity, as explained by Mohammad Mehdi Foroughi et al. Likewise, MOFs are used in a variety of fields, such as photo electronic equipment, medical and biological uses, clean energy storage, and elimination, assimilation, and elimination of hazardous materials from liquids and gases.^7^ Ghaleb A. Husseini et al.^8^ stated that A novel category of porosity composite organic-inorganic materials, MOFs have gained more interest in the last ten years. Applications for MOFs include chemistry, chemical engineering, and materials science. These structures are currently being extensively researched as potentially useful platforms for applications in biology.

Raghu Raman et al.^9^ reported that amidst escalating global energy demands and mounting environmental pressures, sodium-ion batteries (SIBs) have emerged as a compelling alternative to lithium-ion technologies. Leveraging abundant, non-toxic materials, SIBs hold significant promise in advancing the United Nations Sustainable Development Goals (SDGs)-notably SDG 7 (Affordable and Clean Energy), SDG 9 (Industry, Innovation, and Infrastructure), SDG 12 (Responsible Consumption and Production), and SDG 13 (Climate Action).

Min Kim et al.^10^ reported that the degradation of MOFs is directly correlated with the base’s strength and concentration, aligning with our main findings. The compiled material offers important insights that can direct the real-world implementation of Zr-based UiO-66 MOFs in fundamental settings, providing crucial details for their best use in a range of contexts.

**Figure 1:**
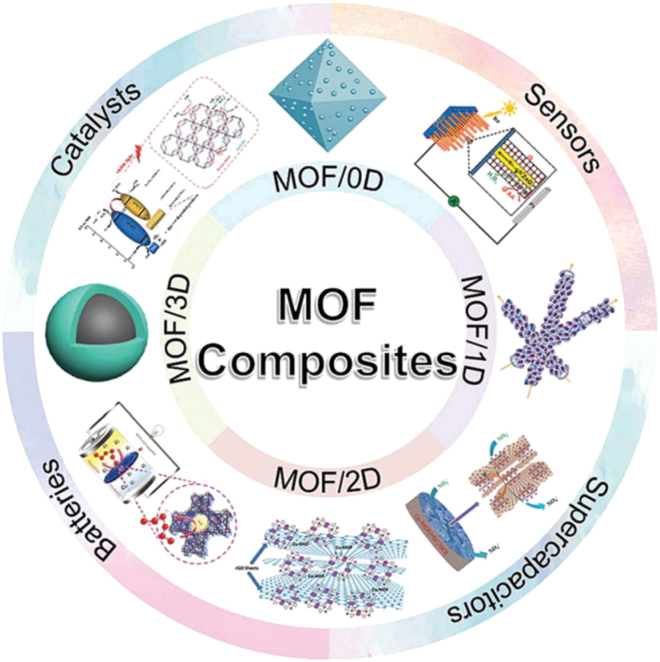
Schematic illustration of MOFs structure and functional applications.^11^ (Copyright 2019 Royal Society of Chemistry)

Huan Pang et al.^11^ elucidated that MOF composites were created to lessen the drawbacks of individual components by combining MOFs with a range of useful materials. Different electrochemical uses of MOF/composites, as summarized, are shown in Figure 1. MOFs can be combined with 0-dimensional components such as quantum dots and nanoparticles, 1-dimensional components such as nanorods, nanotubes, and nanobelts, and 2-dimensional nanosheet components. 3-dimensional MOF materials, such as core–shell and cubic materials, have also been documented. The recent advancements in MOF materials, their production techniques, and their electrical uses in sensors, catalysts, batteries, and supercapacitors are examined in this review based on their dimensions.

MOF composites will be discussed in this section, which is divided into four parts according to the dimension of composite materials, namely, MOF/0D, MOF/1D, MOF/2D, and MOF/3D. 0D materials, with a small size and high surface energy, can enhance the intrinsic functions and properties of MOFs.

Atieh Zamani et al.^12^ stated that the improved Hummer’s method was used to produce three different uniform multilayer self-standing thin films made of placed rGO planes. The samples were studied using Raman spectroscopy, Field Emission Scanning Electron Microscopy (FESEM), 4-point probe measurements, and FTIR to examine their electrical, optical and structural characteristics. The samples of Raman spectra show mild surface imperfections and an elevated O_2_ content of rGOs, while the images from FESEM show a surface that is smooth and sheet-like with few creases. Furthermore, the existence of multi-Stacked layered structures is confirmed by the cross-sectional photographs. The resistance to electricity vs. temperature charts suggest semiconducting properties in the rGOs, since the resistance drops by roughly 0.35–0.65 percent each kelvin in the surrounding temperature range. The samples' FTIR measurement, which was performed between 2.5 and 25 μm in wavelength, shows a notable absorption value of more than 90%. Muhammad Altaf Nazir et al.^13^ introduced that the MOF and carbon activated (MOF@AC) composite, which stands out as a material with a significant area of surface, a variety of pore sizes, and unique characteristics. Highlighting the synthesis methods, essential features, and special qualities of MOF@AC composites is the goal of this review. This thorough analysis mostly concentrates on investigating the various uses of the composite material, which include supercapacitors, energy conversion and storage, photocatalysis, gas storage, and wastewater treatment.

An essential technology for long-term, sustainable energy storage and a clean method of producing hydrogen is electrocatalytic water splitting. Due to their large surface areas, adjustable permeability, ease of compositional modification, and ability to serve as precursors for a wide range of morphologies, metal–organic frameworks have emerged as the most potent multifunctional materials in recent years. MOFs and their derivatives are well identified in this review as an electrocatalyst for the OER.^14^

The production of hydrogen can be possible through only one by-product i.e., O_2,_ and thereby, electricity generated from energy sources for electrocatalytic water splitting is considered to be an important way from the thermodynamic point of view. Deliberate kinetics and overpotentials of OER and HER at anode and cathode limit the use of electrocatalysts for large-scale applications, and, also taking into consideration, the cost and scarcity of noble metal-based materials have enthused researchers to develop earth abundant low cost, outstanding performance and long-term durability metal-based electrocatalysts for both processes.^15^

Hai Bang Truong et al.^16^ reported that significant attention has been drawn to the growing awareness of CO_2_ emissions’ effects as a worldwide issue that is directly related to an increase in global temperature. One crucial strategy to deal with this urgent problem is carbon capture and storage, especially in conjunction with adsorbents. MOFs are intriguing possibilities for carbon dioxide uptake because of their huge surface area, porous nature, and plenty of adsorption sites. Recent advances in MOFs with a variety of adsorption locations, such as Lewis fundamental centers and open metal sites, are covered first in this study. The presentation of many techniques to improve CO_2_ adsorption capacities follows, such as composite construction, post-synthetic alterations, and pore size manipulation. Lastly, the current obstacles and expected opportunities related to the creation of MOF-based nanomaterials for CO_2_ storage are explained. Mustafa A. Alheety et al.^17^ studied creating and evaluating copper ferrite (CuFe_2_O_4_) and graphene oxide (GO) nanocomposites in varying doping ratios (1%–5%) and investigated how well they store hydrogen gas. SEM, TEM, FTIR, and XRD were utilized to describe the produced nanocomposites. The findings demonstrated that whereas GO by itself was unable to store hydrogen at 20°C, the addition of various amounts of copper nanoparticles made of ferrite greatly enhanced the composite materials' capacity to store hydrogen. It was discovered that the ideal proportion of copper ferrite was approximately 3%, and that the amount of ferrite supplied plays a critical part in determining the nanocomposites’ capacity to store hydrogen. In approximately 60 s, the optimal hydrogen storage value of 3.3% was attained at 20°C and 80 bar of pressure.

MOFs have become attractive options for electrochemical applications such as electrocatalysis, batteries, and supercapacitors because of their high surface area, adjustable porosity, and adaptable chemistry.^18^ It is still difficult to turn their potential into workable energy storage solutions, though. Poor intrinsic conductivity, structural instability under cycling circumstances, and synthesis scalability are important problems.^19^ Significant prospects to get beyond these restrictions are presented by recent developments in synthetic techniques, such as the creation of conducting MOFs, composites made of carbonaceous materials, and MOF-derived nanostructures.^20^ This study offers a critical assessment of current trends, material design principles, and future prospects in energy storage research, with a focus on how structural and synthetic tailoring of MOFs can improve their electrochemical performance.

Mei-Yi Tseng et al.^21^ discussed that MOFs, as multifunctional materials, have attracted increasing attention in recent decades due to their unique properties, such as crystalline structure, permanent porosity, high surface area, simple synthetic routes, and self-assembly capabilities. Based on these features, MOF applications are broadly categorized into four main areas: biomedicine, separation, sensing, and catalysis. Our review particularly focuses on emerging research directions, including white light emission, carbon dioxide reduction, water splitting, gas storage, and separation.

Fu-Fa Wu et al.^22^ reported that the growth of energy storage and conversion technologies depends on the creation of sophisticated electrode materials with high specific capacitance and exceptional electrocatalytic efficiency. In this study, a Co_9_S_8_@Ni (OH)_2_ core-shell structured electrode material with exceptional electrochemical characteristics is created using the hydrothermal process. The built supercapacitor achieves an energy density of 60.76 W h kg^−1^ at a considerable power density of 35 280 W kg^−1^, retaining 85.3% of its initial capacity after 15 000 cycles. The material exhibits an impressive specific capacitance of 844.8 C g^−1^ at a current density of 1 A g^−1^. Furthermore, at 10 mA cm^−2^, the material shows remarkable hydrogen evolution reaction (HER) performance, with a Tafel slope of 147.86 mV dec^−1^ and an overpotential of only 92.7 mV. At the same current density, an overpotential of 163.3 mV and a Tafel slope of 50.27 mV dec^−1^ demonstrate the equally impressive performance of the oxygen evolution reaction (OER). These findings highlight the Co_9_S_8_@Ni (OH)_2_ core-shell material as a viable option for improving electrochemical water splitting and supercapacitor technologies.

Yuxi Xu et al.^23^ stated that the Composites made of 3-dimensional graphene (3DG) and MOF have gained growing curiosity about the energy sector due to their unique hierarchical porosity structure and characteristics. When graphene and MOFs are combined, they cannot only successfully get around the restrictions that MOFs have little stability and poor electrical conductivity, but they also stop sheets of graphene from aggregating and reaccumulating. Furthermore, 3DG/MOF hybrid applications in the realm of electrochemistry can be expanded by using them as Multifunctional precursors with tunable derivative composition and structures. The most recent synthesis techniques for 3DG/MOF hybrids and its derivatives are described in this section of the article, along with their uses in electrocatalysis, batteries, and supercapacitors (SCs).

Mustapha Balarabe Idris et al.^24^ discussed that the methodical synthesis of several MOF nanocomposites and their performance in SC. A summary of the present issues and potential paths for MOFs and their carbon-based compound is also provided. MOFs, a new type of crystallized material with pores, have been found by Huan Pang et al.^25^ to have superior qualities such as large areas of surface, adjustable pore size, and various structures that allow them to be used as Supercapacitors, adsorbents, catalysts, and more. An outline of the latest advancements in Materials Based on MOF/Graphene is given in the study, with particular attention paid to their methods of preparation and uses in the filtration, storage, and separation of gases and water, detectors for chemicals, super capacitors, batteries, and catalysts. Graphene Oxide linked with organic-inorganic hybrid components, ranging from complexes of 0D coordination MOFs in three dimensions, presents encouraging opportunities, according to Christian Serre et al*.*^26^ The purpose of this evaluation is to offer a global synopsis of various synthetic routes that have been implemented in the literature, along with the descriptions and uses of these systems.

## Techniques for creating functional metal-organic frameworks structures

• Hydrothermal synthesis and solvothermal synthesis: The most popular techniques allow for great crystallinity and morphological control. The pore structure and surface area can be fine-tuned by adjusting the temperature, solvent systems, and metal/ligand ratios.^27^
• Ultrasonic and microwave-assisted synthesis: These provide energy efficiency and quick reaction kinetics, frequently resulting in MOFs with smaller particle sizes and improved electrochemical performance.^28^
• Template-assisted methods: Hierarchical porosity, which promotes ion transport and increases the accessibility of electroactive materials, is introduced using both hard and soft templates (such as SiO_2_ spheres and surfactants).^29^
• Modification after synthesis: An adaptable method for enhancing conductivity and electrochemical stability by adding functional groups, heteroatoms, or redox-active sites to the MOF framework.^30^

**Figure 2:**
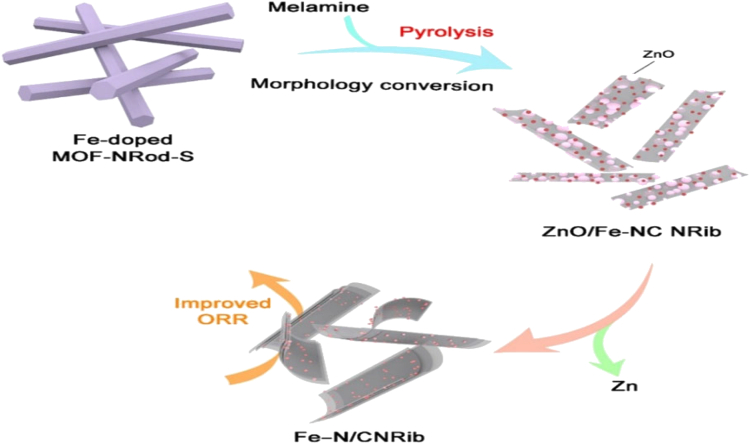
Methods for creating functional 2D CNRibs synthetically from tiny 1D MOF NRods.^31^ (This figure has been published in *CCS Chemistry* [2022]; [One-Step Synthesis of Ultrathin Carbon Nanoribbons from Metal–Organic Framework Nanorods for Oxygen Reduction and Zinc–Air Batteries] is available online at [https://doi.org/10.31635/ccschem.021.202101160])

Qiang Xu et al.^31^ reported that the Energy-related applications heavily rely on 2-dimensional (2D) Carbon Nanostructures; however, it is quite uncommon to find simple and effective methods for creating these nanostructures. A 1-step decomposition of 1-Dimensional MOF NRods has been used to deliberately create ultrathin carbon nanoribbons (CNRibs), which have a thickness ranging from 2 to 6 nm and an overall length of more than 100 nm. Porous nanostructures of carbon in 1D or 2D topologies can be created by adjusting the dimensions of MOF NRods as shown in Figure 2. Profound understanding of the structural transition from one-dimensional to two-dimensional morphology, offering a productive method for creating Nanomaterials with low dimensions that have tunable electrochemical and morphological application functions.

## Metal-organic frameworks-based porous nanostructures in supercapacitor technology

**Figure 3:**
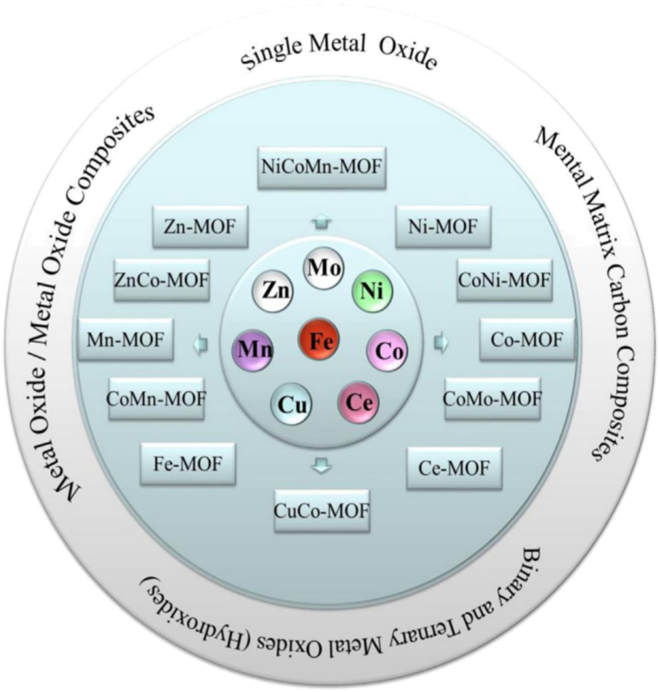
Classification of MOFs based on single, binary, and ternary metal centers (Zn, Mo, Ni, Fe, Co, Ce, Cu, Mn) and their composite types: single metal oxides, metal oxide composites, carbon matrix composites, and hydroxides.^32^ (Copyright 2020 Elsevier)

Jiangfeng Li et al.^32^ stated that the area of supercapacitors favors MOFs as an emerging class of porosity polymers with coordination because of their highly devisable topologies, Super-Large area of surface, controllable porosity, and variable composition. A variety of MOF-Derived Nano porous materials, as shown in the Figure 3 later in discussion, such as metallic matrix carbon hybrids and mono-as well as binary and ternary metals, have been used recently in supercapacitors. The current development of MOF-derived Nano porous substances, together with their synthesis process, shape, and electrochemical properties, has been covered in that review. Effective methods for materials formed from MOFs have also been suggested, and the trend in utilization and development has been examined.

### Multifunctional nanomaterials for next-generation supercapacitors

**Figure 4:**
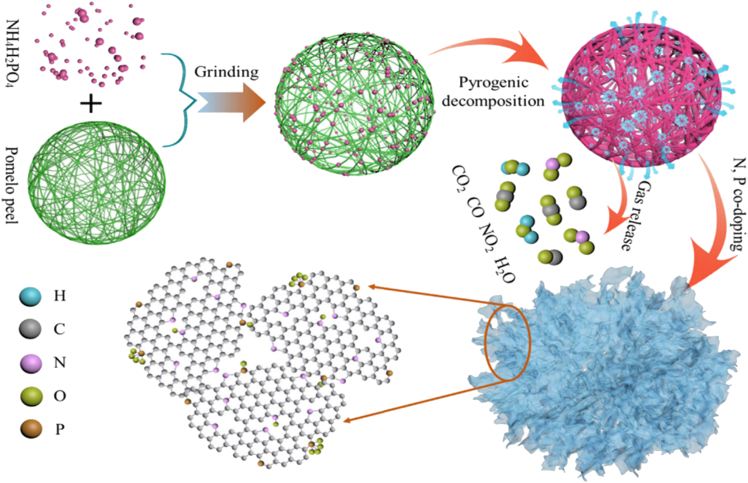
Representation of the NPCN synthesis process.^33^ (Copyright 2022 Elsevier)

Lishan Jia et al.^33^ synthesized N, P Co-doped Porous-Carbon Nanosheets (NPCNs) as shown in the Figure 4 were synthesized from the peel of pomelo utilizing NH_4_H_2_PO_4_ as a co-dopant and activator in order to employ them as supercapacitors with notable performance. Thus, the ideal sample has high-rate Capacity (82% of capacitance preservation at 20 A/g) and high particular capacitance (314 ± 2.6 F g^−1^). With a high energy density of 36 ± 1.5 W h kg^−1^ and a power density of 1000 W kg^−1^, with 99% preservation after 10,000 cycles, NPCNs-750 was also used in a chiral supercapacitor (NPCNs-750//NPCNs-750 SSC) with 2 M Li_2_SO_4_ electrolyte. Excellent performance materials for supercapacitor electrodes can be designed and prepared using this economical and free of pollutants technology, resulting in several outstanding results.

This is a method for doing heteroatom doping, activation, and carbonization all at once. The non-corrosive NH_4_H_2_PO_4_ was utilized as the co-dopant of N, the carbon source was the leftover pomelo peel, and the activator of carbon components, as well as P atoms. N, P Co-doped biomass carbon nanosheets (NPCNs) were effectively made with the fewest possible extra ingredients and processes. The hierarchical pore structure of the resulting NPCNs facilitates the quick implantation and diffusion of electrolyte ions. Furthermore, Co-Doping N and P atoms can increase the number of active centers in carbon-based materials, enhancing the materials’ Electrochemical properties. The best sample, NPCNs-750, shows outstanding electrochemical properties, including high energy density, strong rate performance, greater capacitance, and superior cycle stability, when utilized as a supercapacitor electrode material. Consequently, this effort will pave the way for the sustainable production of promising biomass-based energy storage materials.

Zuozhao Zhai et al.^34^ given that the most widely utilized material for electrodes for supercapacitors is carbon, so study on carbon materials is important for the development of supercapacitors. The energy storing methods of supercapacitors and the widely utilized carbon electrode components are presented in this article. The energy preservation mechanism comprises widely utilized energy storing models, as well as the validation and thorough comprehension of these models through the application of *in-situ* technologies and molecular dynamic simulation. The most commonly employed carbon materials and their uses in supercapacitors are introduced in the section on carbon electrode materials. The Co_3_O_4_-Ni-Metal organic frameworks blend material is effectively manufactured by Shiping Luo et al.^35^ using a Solvent-Thermal technique. It was then mixed with acidified carbon nanotubes with multiple walls; The Co_3_O_4_-Ni-MOF/MWCNTs hybrid that was produced was used within a supercapacitor and examined using XRD, Infrared Spectroscopy using the FTIR, and SEM. Therefore, regarding the materials used in supercapacitors, Co_3_O_4_-Ni-MOF/MWCNTs mixed has a comparatively excellent application possibility as an electrode material. According to the experimental results, Co_3_O_4_-Ni-MOF material exhibits superior electrochemical performance when metal oxide and Ni-MOF compounds have a Molar ratio of 1:1. Because of the 1:1 ratio of mass between MWCNTs and Co_3_O_4_-Ni-MOF, the hybrid Co_3_O_4_-Ni-MOF/MWCNTs shows improved supercapacitor effectiveness, a value for the least Rct, and enhanced stability of cyclic capacitance. Nashwa M. Yousif et al.^36^ explained that the process of creating adjustable electrodes for supercapacitors’ cathodes using EVA thermoplastic material film, poly aniline, and carbon. In this case, EVA/PANI@CNT adjustable films were created as super capacitor cathode electrodes with outstanding electrochemical properties and elasticity by combining EVA, the pseudo-capacitance of PANI, and CNTs’ capacity to carry charges. Carbon nanotubes/polyaniline (PANI@CNT) composites with Ethylene-Vinyl Acetate, which uses radiation from an electron beam at 100 kGy. Finally, they proved that enhancing the electrically charged material/current collection interface concentration can accelerate electron transit and is a potent tactic to strengthen the electrically capacitive characteristics of supercapacitors.

### Real-world application potential of Ni/co-metal-organic frameworks-rGO supercapacitors

The dual Ni/Co-MOF- rGO nanocomposite’s One-pot Co-amalgamation and previously unreported in their study, Mir F. Mousavi et al.^37^ described a water-stable Co-MOF-based nickel-based MOF (Ni-MOF) in the same reaction vessel. 860 F/g of significant specific capacitance at 1.0 A/g is demonstrated by the rGO-MOF-Ni/Co nanocomposite. The asymmetrical triggered Carbon//Ni/Co-MOF-rGO Component has a long lifespan of cycle (91.6% capacity persistence following 6000 cycles of charge and discharge at 1 A/g) and can deliver a particular energy level of 850 W/kg at 72.8 Wh/kg while maintaining 15.1 Wh/kg at the greater particular power of 42.5 kW/kg. A blue-colored light emitting diode (LED) is powered by two 1.0 cm^2^ devices linked in series for 25 min, an LED with green color for 25 min, along with a red LED for over 140 min. They also run a clock for approximately 2 h. This shows that one extremely promising material is a nanocomposite, which is active regarding real-world uses.

The electrochemical tests were conducted after the composite of Ni-MOF and Co-MOF/rGO with weight percentage ratios of 25:25:50 was activated. Compared to the previously prepared Co-MOF and Ni-MOF electrode, the final composite of the Ni/Co-MOF/rGO exhibits Cs of 860 F/g at a constant current density of 1.0 A/g. These enhancements in electrochemical performance could be brought about by rGO’s extremely porous nanosheets. Kim et al.^38^ used the hydrothermal technique to create Ni-based MOFs nanocomposites with rGO for the Sc application. For Sc testing, they employed the Ni-MOF/rGO electrode’s constructed cubic structure as an operational material, and they found that the composite had greater values than the other electrodes. At a steady current density of 1 A/g in the 6 M KOH, the Cs of the Ni-MOF/rGO electrode was 1154.4 F/g, with improved retention up to 90% at 3000 cycles. Beka et al.^39^ reported Perhaps as a result of the beneficial synergistic effect between the Ni-Co based MOFs and the varying concentration of rGO electrode material, the optimized Ni-Co based MOFs/rGO-2 prepared at a 10 mg concentration of the rGO composite with Ni-Co based MOFs exhibits exceptional electrochemical performance with a higher Cs of 1553 F/g at a lower current density of 1 A/g. The Cs of the Ni-Co-based MOFs electrode is 901 F/g at an electrode current density of 1 A/g. After 5000 cycles, the optimized Ni-Co-MOFs/rGO composite electrode demonstrated improved stability with 83.6%. Following electrochemical property testing, they created asymmetric devices with an impressive 44 Wh/kg energy density and 3168 W/kg power density.

### Innovative metal-organic framework architectures and hybrid materials for supercapacitor applications

SBUs, or secondary building units, constitute the turning points which enable enormous fundamental stability variety, thermodynamic durability, as well as architectural and mechanical the durability for demonstrating materials as needed with planned layouts in the synthetic creation of MOFs via more powerful bonding between their part metals and Organic bonds, according to Mohanty et al*.*^40^ This article emphasizes the application of MOFs, particularly 3-dimensional MOFs, which is a platform of supercapacitor applications. Young et al.^41^ described a simple one-step process that uses the NiCo-MOF-74 bimetallic as the beginning prelude to create new Materials that are hybrid with pores. Graphitic carbon/Ni_x_Co_1−x_ Composites (also referred to as NC-800) are created when the NiCo-MOF-74 bimetallic particles are subsequently carbonized at 800°C in a Nitrogen environment. On the other hand, NiCo-MOF-74 can be heat treated at 350°C in air (referred to as NC-350) to produce Ni_x_Co_1−x_/Ni_x_Co_1−x_O blends (with a dash amount of C). NC-800 & NC-350 have high particular capacitances of 715 and 513 F g^−1^ at an elevated current concentration of 1 A g^−1^, respectively, when evaluated as supercapacitor electrode materials. Additionally, after 2,500 cycles, there is no discernible reduction in these composite materials’ specific capacitance, indicating nice steadiness when cycling. The MOF’s have a high degree of tunability, which includes favourable porosity characteristics, Programmable Chemical Compositions, controlled crystal structures, and customizable geometric morphologies. This has caused them to be widely used in supercapacitor applications, according to Xu et al*.*^42^ The latest research on MOF-based applications for supercapacitors is discussed in detail in this review.

Teng et al.^43^ Describes that the highly capacitive active material, which is made up of polyaniline (PANI) nanoparticles and manganese dioxide (MnO_2_) nanowires that are developed consistently on the matched microchannels’ porous wall (CW@PANI and CW@MnO_2_), is housed in a three-dimensional carbonized timber matrix that functions as a porous host and a surface area that is huge current collector. A high capacitance of the region of 1 mA cm^−2^ at 729 mF cm^−2^ (369 F g^−1^ at 0.5 A g^−1^) may be delivered by the electrode on the positive side due to the electrolytic interchange reactive sites that occur. An elevated area capacitance of 1721 mF cm^−2^ at 1 mA cm^−2^ (848 F g^−1^ at 0.5 A g^−1^) is delivered by the negative electrode. Two electrodes’ real capacitance is higher than that of the majority of the electrodes of supercapacitors that have been reported. Furthermore, a significant work functional distinction between the electrodes produced a 2 V broad voltage window, which allows the combined CW@MnO2//CW@PANI AASC to attain a suitable energy density (170.84 μm Wh cm^−2^ at a power density of 0.5 mW cm^−2^).

### Nanostructured hybrid materials and conductive polymers for high-energy supercapacitors

Aligned conductive polymeric arrays of nanowires and their combinations Nano-Carbon materials have the potential to made using Electrical polymerization lacking a template or *in situ* chemically polymerization, which are regulated by the polymerization process’s nucleation and growth phases (Wang et al*.*^44^). The outstanding flexibility, excellent Conductive polymers’ enormous capacitance and processability are used in these Supercapacitor Prototypes, effectively extending their use in a variety of scenarios as well as even for an intricate system of the integration of several electronic gadgets. Dsoke et al.^45^ note that materials that exhibit reversible redox action, or battery-like characteristics, can also become Pseudocapacitive by nanosizing. The potential for concurrent delivery of high energy and high power is another benefit of hybridization using electrodes of the capacitive and Faradaic types at the device level. Lastly, by improving the electrode’s charge storage transit, nanosizing can increase the conductivity of electricity and reaction kinetics. All of these topics are covered in detail and reviewed in this chapter. Tabassum et al.^46^ discovered that metal nano-confinement within CNTs not only enhances the CNTs’ local surroundings but also stops metal aggregation and leaching. Recent developments in creating sophisticated nano-confinement techniques for various useful nanoparticles, including metals, oxides of metals, metal sulphides, metal phosphides, and metal carbides, are compiled in this critical review. Lastly, the difficulties and potential. There is a discussion of M@CNTs for electrolytic conversion and storage of energy systems.

The insulating property of MOFs is frequently acknowledged as an obstacle in the extending of their uses, particularly in electronic areas, according to Sundriyal et al*.*^47^ To increase MOFs’ potential for such applications, a variety of conductive or useful substances have been combined or intercalated with them in light of these constraints (e.g., batteries that are rechargeable, optical electronics, and Supercapacitors). The use of several types of electrolytes (such as water-based, organic farming, Ionized liquids and solid-state devices, Redox electrolytes, as well as effects of the advancement of supercapacitors based on MOF has also been covered. Five novel Frameworks for Crystalline Zeolitic Imidazolates (ZIFs), ZIF-78 to 82, were synthesized for Zinc (II) Nitrates and their combinations, comprising five different functionalized imidazoles and 2-nitroimidazole. van Essen et al.^48^ stated that Matrimid matrices were used to methodically clarify the consequences of the different pore volume, pore aperture, pore diameter, and capabilities of the ZIFs on the mixed gas CO_2_/CH_4_ and CO_2_/N_2_ MMM permeation. These 3- isoreticular gmelinite (GME) ZIFs, namely ZIF-68, 69, and 78, are distinguished by their partially distinct imidazolate linkers. Without sacrificing selectivity, the addition of ZIFs to Matrimid raises the CO_2_ permeability across for both CO_2_/N_2_ and CO_2_/CH_4_ input, all MMMs combinations. The 20-weight percent ZIF-68 MMM showed the greatest improvement in carbon dioxide permeability, exceeding the permeability of traditional ZIF-8/Matrimid MMMs by increasing CO_2_ permeability by122% for CO_2_/CH_4_ feed and 116% for CO_2_/N_2_ feed in comparison to Matrimid.

## Distinct CO_2_ mobility in UTSA-74: insights from a framework isomer of MOF-74

**Figure 5:**
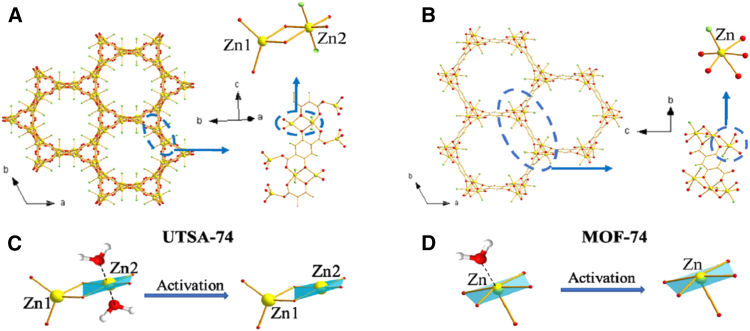
Crystal structures and activation processes of UTSA-74 and MOF-74 Structure of UTSA-74 (A) and MOF-74-Zn (B); (C) Illustration of binuclear Zn cluster in as-made UTSA-74 and the formation of two OMSs on Zn_2_; (D) Illustration of the formation of OMS in MOF-74-Zn. (Adapted with permission from Li et al.^49^).

Li et al.^49^ investigated that MOF UTSA-74, the structure isomer of the renowned MOF-74, is special in that it has 2 OMSs on one metal ion when it is activated, as shown in Figures 5A–5D. It offered a singular chance to investigate the kinetics of CO_2_ molecules deposited on these OMSs. This study investigated MOF UTSA-74’s carbon dioxide adsorptive characteristics, paying special emphasis to the kinetics of the adsorbed CO_2_ by these OMSs. In particular, 13CO_2_ action in UTSA-74 at various loadings was directly observed by fluctuating temperature 13C stable SSNMR measurements. Some CO_2_ molecules bounce between 3 OMSs from 3 distinct Zn atoms within the channel’s cross-section at low loadings. There are others who alternate between the two nearby OMSs. Two-site hopping continues at heavy loads, but the three-site jump has stopped. The special Zn binding environment in UTSA-74 is the cause of the dynamical behavior of CO_2_. CO_2_ was shown to be less mobile in UTSA-74 compared to MOF-74-Zn, its framework isomer.

The Zn-MOF-74/CPO-27-Zn isomer, a novel metal-organic framework Zn2(H2O) (dobdc)·0.5(H2O) (UTSA-74, H4dobdc = 2,5-dioxido-1,4-benzenedicarboxylic acid), has been synthesized and structurally described. It features one-dimensional channels of roughly 8.0 Å and a unique four coordinated fgl structure. There are two distinct Zn (2+) sites within the binuclear secondary building units in UTSA-74, one of which (Zn1) is in a tetrahedral coordination geometry and the other (Zn2) is in an octahedral coordination geometry. This is in contrast to the metal sites in the well-known MOF-74 with a rod-packing structure, where each of them is in a five-coordinate square pyramidal coordination geometry.^50^ Bueken et al.^51^ reported that the water-induced conversion of the metal-organic framework UTSA-74 to its polymorph MOF-74(Zn) of [Zn2(dobdc)] (dobdc = 2,5-dioxidobenzene-1,4-dicarboxylate), in contrast to a earlier study on UTSA-74’s stability in these circumstances. Time-resolved *in situ* X-ray diffraction was used to study this dissolution-recrystallization process, and the Gualtieri crystallization model was used to examine its kinetics.

The surface areas and pore volumes of MOFs enable massive loading and rapid release, making them promising drug carriers. In order to administer ibuprofen, this study examined two biocompatible MOFs: Zn MOF-74 and UTSA-74. The MOFs were impregnated with 30, 50, and 80 weight percent ibuprofen in order to examine the impact of drug loading. N2 physisorption, XRD, and SEM were used to characterize the materials. According to SEM, the MOF structures were preserved at 30 weight percent ibuprofen; however, with 50–80 weight percent loading, they agglomerated as the medication attached to the particles and accumulated on the surface. The Zn MOF-74 samples’ surface area declined with ibuprofen loading in the physisorption experiments, reaching zero at 80 weight percent.^52^

## Synthesis methods of metal–organic frameworks

MOFs represent an emerging class of Crystalline Porous Hybrid Materials whose structure, porosity, and functionality can be precisely tuned. They are well suited for use in energy storage, gas separation, environmental remediation, and catalysis due to their large surface area, carefully designed pore structures, and variety of synthesis methods, including hydrothermal, microwave, electrochemical, and mechanochemical approaches. However, obstacles to their widespread use include structural instability, poor electronic conductivity, and insufficient photocatalytic efficacy.^53^

**Figure 6:**
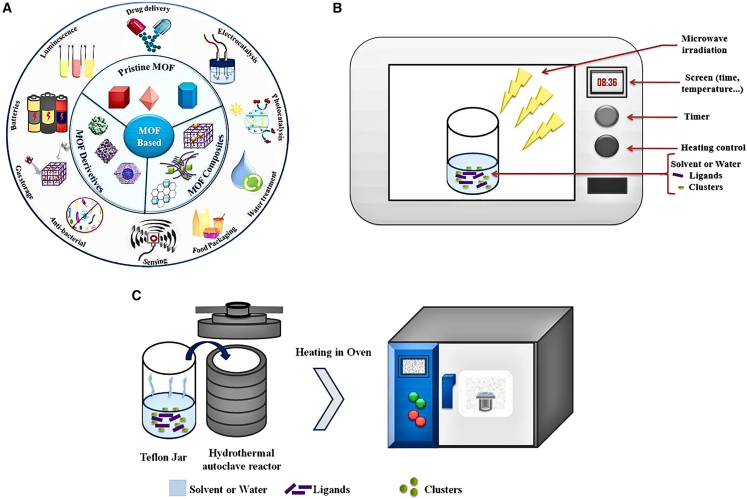
Synthesis and applications of metal–organic frameworks (MOFs) (A) Summary of pure and MOF composite properties and their applications. (B) Microwave technique of MOF-synthesis (C) Solvothermal/hydrothermal process of MOF-preparation (Adapted with permission from Zeggai et al.^54^ and Copyright 2025 Elsevier).

Zeggai et al.^54^ described that the structure, porosity, and usefulness of MOFs, a novel type of crystal porous composite materials, can be precisely tuned. Their large area of surface, carefully designed pore structures, and variety of synthesis methods (Figures 6A–6C) (including electrochemical, hydrothermal, mechanochemical, and microwave approaches) put them in a prominent position for use in the separation of gases, storage of energy, restoration of the environment, and catalysis. However, their widespread use is hampered by problems such as poor photocatalytic efficiency, subpar electrical conductivity, and instability in the structure. Significant progress has been made thanks to novel approaches such as composite development, heteroatom doping, and heteroatom defect engineering with hybrid properties. For example, photocatalytic hydrogen evolution is increased by 40% in Ti-doped MOFs and five times in electrically conducting Ni-MOF hybrids.

Metal–organic frameworks (MOFs) feature rich chemistry, ordered micro-/mesoporous structure, and uniformly distributed active sites, offering great scope for electrochemical energy storage (EES) applications. Given the particular importance of porosity for charge transport and catalysis, a critical assessment of its design, formation, and engineering is needed for the development and optimization of EES devices. Such efforts can be realized via the design of reticular chemistry, multiscale pore engineering, synthesis methodologies, and post-synthesis treatment, which remarkably expand the scope of applications.^55^

Tajik et al. have created MOFs specifically for electrochemical applications. MOFs are an adaptable type of porous material with large areas of specific surface, manageable structures, and tunable pores. In recent years, MOFs have been recognized as a promising platform in the field of electrochemistry. After describing the latest applications of MOFs and their composites in electrochemical sensing, electrocatalysis, and electrochemical energy storage devices (such as batteries and supercapacitors), we provide a summary of the issues and perspectives that still need to be addressed for MOF-based materials in these fields^56^ provided different synthesis methods and their applications in Table 1.

**Table 1:** Different production methods, MOF materials, and their applications

S. No	Main Material (SC Component/MOF-based)	Method	Ligand	Metals	Material Composition	Aim	Application	Reference
1	Indium-based MOFs (InOF-1, MIL-60, MIL-68(In))	Solvothermal synthesis (MOF design and optimization)	1,4-benzenedicarboxylic acid (BDC) derivatives (assumed typical for these MOFs)	Indium (In^3+^)	MIL-60 with 6.5 Å pores and high lattice oxygen density (19.97 nm^−3^)	To achieve synergistic Li^+^ conduction by combining anion immobilization (via pore exclusion) and cation hopping (via lattice oxygen sites)	Solid-state lithium-metal batteries	Xu et al.^57^
2	Defect-engineered Hf-MOF-based quasi-solid-state electrolyte (Hf-MOF-QSSE)	Defect engineering to enhance open metal site (OMS) density and optimize pore microenvironment	carboxylate- or nitrogen-based ligands in Hf-MOFs)	Hafnium (Hf)	Defect-rich Hf-MOF incorporated into QSSE with optimized OMSs for improved Li^+^ conduction	To enhance Li^+^ salt dissociation and ionic conductivity through defect-engineered OMSs	High-energy-density lithium batteries, tested in ∗∗Li	Wang et al.^58^
3	MOF@GF (Metal–Organic Framework modified Glass Fiber) reinforced Quasi-solid Composite Polymer Electrolyte (QCPE)	*In situ* 3D self-assembly of MOF on glass fiber, followed by soaking in liquid electrolyte	carboxylates or imidazolates	Zr, Al, or In	Polyelectrolyte matrix + 3D MOF@GF + small amount of liquid electrolyte → forming Quasi-solid composite polymer electrolyte (QCPE)	To develop a mechanically strong and ionically conductive composite electrolyte for use in structural lithium batteries	Structural lithium-metal batteries	Wang et al.^59^
4	3D Lattice-Oxygen MOF (MIL-60, InOF-1, MIL-68(In))	Interlayer engineering for solid-state batteries	Benzene-1,3,5-tricarboxylic acid (BTC) or terephthalic acid (based on MIL-type MOFs)	Indium (In^3+^)	MOF-based interlayer: porous MIL-60 (In) integrated between cathode and solid-state electrolyte	Improve Li^+^ conductivity, block TFSI^−^ anions, enable fast charging with high stability	Quasi-solid-state lithium-metal batteries (LMBs)	Muhammad et al.^60^
5	Graphite–Silicon Blended Composite Anode (Note: This is not a MOF-based system, but a mechanically modeled silicon/graphite blend)	Multiphysics electrochemical–mechanical–thermal modeling (simulation-based study)	Nil	Anode materials: graphite and silicon	Composite of graphite and silicon particles (volume ratio varied, e.g., 4% silicon)	To analyze and model the effects of mechanical stress, Li^+^ diffusion, and silicon content on hysteresis, voltage behavior, and heat generation in anodes	Cylindrical-type lithium-ion batteries	Du et al.^61^
6	MOF-filled Composite Polymer Electrolyte (CPE)	*In situ* composite fabrication (MOF + polyurethane + glass fiber)	oxygen/nitrogen donor ligands	Not specified	Highly crosslinked polyurethane + glass fiber + porous MOF (for ionic conductivity)	Enhance ionic conductivity and mechanical strength of polymer electrolytes	Solid-state lithium metal batteries	Li et al.^62^

## Versatility of metal–organic frameworks in various fields

### Combining functional devices with metal–organic frameworks

Stavila et al.^63^ outlined the existing status regarding MOF studies in the field’s electronic devices in nature, optoelectronic, as well as sensors and concentrated our focus on the fundamental prerequisites along with the structural components required to construct MOF-Oriented technologies. We provide an overview of various techniques for developing active MOFs, assembling and integrating MOFs with instrument hardware, and creating hybrid material systems that combine MOFs with other materials. Zou et al.^64^ constructed that the conversion of 2-dimensional nanosheets into 3-dimensional regular patterns maximizes the potential of 2-dimensional components in uses such as catalysis and simplifies mass transfer. The process entails the epitaxial development of individual crystallographic MOF nanosheets on a cubic MOF core, with the shell having a naturally undesirable aspect exposure. The nanosheets additionally create a single crystal orthogonally organized framework, although having two typical forms and crystalline orientations. It is possible to precisely control the density and dimension of the Core-Shell-Structured nanosheets hybrid MOF.

## Basic insight into supercapacitor types

### Electric double layer capacitance and pseudo capacitors

Saleem et al.^65^ reported that the one kind of storage of energy device is a supercapacitor, because of its unique reaction mechanisms, it has a huge specific capacitance, excellent cycle stability, high power density, and capacity for rate.

Pseudo capacitance and Electric double layer capacitance (EDLC) were the 2- main storage of charge methods that supercapacitors in trust. The arrangement of ions at the contact between the electrode and electrolyte is a component for EDLC type charge storage. High power density is attained in this technique because of the quick ions are adsorbed and desorbed at the electrolyte-electrode contact. Furthermore, just a movement and arrangement of ions takes place during this charge storage mechanism; no chemical reaction takes place.

**Figure 7:**
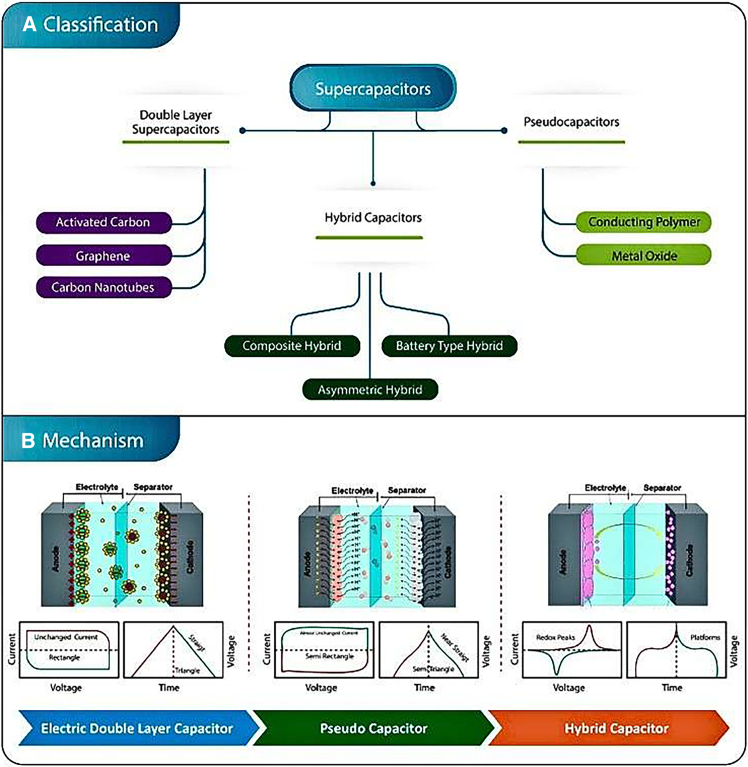
A classification and mechanistic overview of supercapacitors (A) Supercapacitor classification scheme (B) Mechanism of different types of supercapacitors [With permission of ref.^10^ copyrights 2020, coordinationchemistry reviews]. [With permission of ref.^3^ copyrights 2017, National Science Reviews]. (Adapted with permission from Saleem et al.^65^ Copyright 2024 Elsevier).

The pseudocapacitive mechanism, on the other hand, produces high energy density by following the Electrochemical Surface Faradaic Oxidative and Reducing Reaction in conjunction with ion adsorption-desorption, which is similar to that of batteries. On the other hand, for pseudocapacitive materials, the Cyclic Voltammetry profile reveals capacitive properties. Because of their hybrid capacitive and faradaic reaction mechanism, they are referred to as pseudo-capacitance. CNTs, Graphene, carbon aerogel, multiwall Carbon Nanotubes, rGO, and other carbon-based materials typically exhibit EDLC-type charge storage mechanisms. Materials made of carbon are appropriate for this process of charge storage because of their large strong stability in structure, excellent conductivity, and a particular area of surface. This is because the electrode’s material’s active surface area significantly affects the electrochemical execution of EDLC type materials. Nevertheless, these materials possess a reduced energy density because of their inferior specific capacitance. See in Figures 7A and 7B depict the many types of supercapacitors together with their Classification and Mechanism.

### Hybrid supercapacitors

Due to their favorable energy and power densities, hybrid supercapacitor technologies—built utilizing both battery-like and capacitive electrodes-have been embraced as promising devices for energy storage (Wang et al.,^66^). To increase the total energy capacity for hybrid supercapacitor storage, it is essential to design and fabricate capacitive and battery-like high specific capacitances/capacitances, increased rate efficiency, and the required endurance are characteristics of electrode materials.

## Metal–organic framework supercapacitor materials

Ajdari et al.^67^ looked into how the world’s energy needs, High carbon dioxide emissions, the quick exhaustion of fossil fuels, and the seriousness of sustainable energy have all worked to encourage the development of High-Performing, environmentally friendly storage of energy systems. Currently, when developing an effective storage of energy system, factors such as great energy and density of power, extended life of High carbon dioxide emissions, the quick depletion of fossil fuels, as well as the seriousness of sustainable energy promises have been considered. Ultra-Flexibility, environmental friendliness, low weight, safety, and dependability are also considered. With their desirable Electrochemical advantages—such as a high density of power, extended cycle life, fast Rate of charge-discharge, and highly sought-after commercial attributes —supercapacitors are developing at a rapid and significant rate. Here, we reviewed submitted articles on the usage of MOFs in SCs and the latest advancements in the electrode and electrolyte components used in super capacitive behavior. Technical results show that MOF-Based SCs commonly use Cu_3_(HITP)_2_, Ni_3_(HITP)_2_, MOF-74, MOF-5, UiO-66/67, MIL-Based, and ZIF-Series. In addition to being very dependable documents—much more so than articles—patents are also expensive to file. This research, in our opinion, offers a fresh perspective on MOF-based SCs for potential future electrochemical considerations that could result in better-performing devices.

## Methods for making metal–organic framework and the useful materials they yield

### Pristine metal–organic frameworks

Pure Metal Organic Frameworks composition and structure have a significant influence upon their electrochemical properties, according to Tian et al*.*^68^ The architecture of MOFs, which consists of frameworks as well as porous structures, impacts their Specific Area of Surface, conductivity of electricity, Structural stability, electrical conductivity, and the transport channel. These elements are intimately related to the MOFs’ transport of ions and electrons capabilities. The number of active sites and electrochemical activity are also based on the composition, especially the metal Center, which demonstrates the pseudo capacitor behavior of Metal Organic Frameworks. This section will cover the synthetic techniques for modifying the structures and compositions of virgin MOFs.

### Metal–organic framework composites

MOFs are an emerging class of coordination polymer that is transparent, according to Peng et al*.*^69^ They stand out among polymer substances and have garnered significant notice due to their exceptional qualities, which include Ha high level of porosity, a large area of surface, and adjustable structure. However, MOFs’ limited electrical conductivity and small micropores have limited their use. Here in this evaluation, MOF composites, their synthesis techniques, and their electrochemical uses in detectors, catalysts, super capacitors, batteries, and super capacitors are currently addressed relative to their dimensions.

### Metal–organic framework conversion into derivatives

Metal–organic frameworks are currently considered for the best electrode components as well as predecessors for the transformation and preservation of electrochemical energy (EESC), based on He et al*.*^70^ This is because of their huge Particular Surface Areas, very adjustable porosity, plenty of active sites, and a variety of ion node and organic linker options. The synthesis techniques, structural and morphological controls, and performance benefits in specific applications of MOF-Derived and MOF-Based powdered the materials have been extensively examined. Lastly, additional viewpoints on these workable options for standalone EESC MOF-based/derived electrodes, as well as insights into the difficulties currently confronted nevertheless, both bindings and additions would be needed to create post-processed electrodes in order to use them for energy applications. This would essentially remove part of the websites that are operational and diminish the higher-up benefits of the substances made from MOFs. The structural characteristics and manufacturing processes of independent MOF-based or derived electrodes are thoroughly covered in this research. The most recent developments in standalone MOF-derived/based electrodes, ranging from devices for storage of electrochemical energy to electrocatalysis, are then outlined with the goal of offering a fresh set of guidelines to encourage their continued advancement in commercial applications and expansion of production.

### Metal–organic framework derived hollow metal oxide

**Figure 8:**
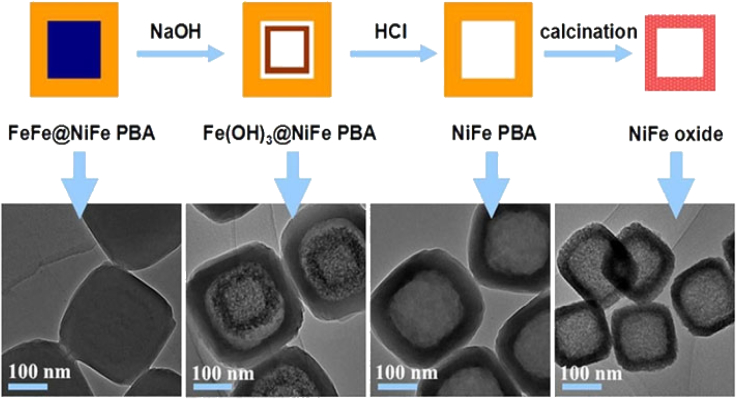
Schematic and TEM images show the synthesis route from FeFe@NiFe PBA to NiFe oxide via NaOH and HCl treatment followed by calcination.^71^ (Copyright 2016 Royal Society of Chemistry)

Cai et al.^71^ showed how to create hollow and intricate MOF structures as well as peculiar MOF composites using a chemical transformation technique. This approach could cleverly get around the problem brought by the potential mismatch in the lattice between other functional materials and MOFs. The effective synthesis of a number of distinct MOF Hollow, MOF Composites, MOF@MOF Core-Shell Structures, and complicated Hollow structures is impressive. In contrast to their simple nanobox counterparts, NiFe oxide, as shown in Figure 8, is a complicated nanostructure inside a box that is hollow, for instance, and was assessed as a lithium-ion battery anode component and shows improved performance in electrochemistry.

The practical efficacy of supercapacitors and batteries (SCs) is largely dependent on the electrode materials chosen. Outstanding characteristics such as high reversible capacity, cycle performance, and exceptional rate capability have been shown by electrode materials made from MO composites and produced from MOFs. These characteristics make them especially appealing options for electrode materials.

## Photovoltaic application of metal–organic frameworks

The requirement for Hydrogen Utilization and CO_2_ reduction has caused a substantial amount of interest in the field of research disciplines for the storage of gases with separation during the past 20 years, as demonstrated by Chueh et al.^72^ in Metal–organic framework (MOF) materials. Furthermore, because MOF materials have some special qualities that allow for improved performance and stability in photovoltaic systems, many MOFs that are functional MOFs have lately been used as well as explored in the photonic sector. To illustrate the potential of MOFs in solar cell apparatus, such as Solar energy cells sensitive to dyes, organic–inorganic solar cells that are hybrid made of perovskite and natural solar panels, this article focuses on providing a thorough overview of previous advancements in these uses.

## Stabilization of metal–organic frameworks

MOFs have been considered one of the most significant material types in recent years, according to Kirchon et al*.*^73^ The MOF family is constantly growing as a result of the pairing of different Ligands and metal Clusters organized in a wide range of designs. Structurally diverse MOFs are continually being developed each year. The use of these materials has significantly increased due to the wide range of structural variations available in MOFs. This review’s scope will include the basic elements of MOF design and synthesis as well as real-world applications pertaining to their durability and derivative structures. There will also be a discussion of new developments in MOF development. These trends will include MOF composites, multicomponent MOFs, and defect formation in MOFs. We all also look into the crucial structure-property-application relationship for MOFs. All things considered, this review sheds light on MOFs’ current structures as well as their developing features.

## Conclusions

The vast surface area, adjustable permeability, and variety in chemical functions of MOFs have made them extremely encouraging as electrode components for supercapacitors. In addition to highlighting MOFs’ benefits in energy storage applications, the review has emphasized the critical elements influencing MOFs and their composites’ structural and electrochemical properties. Nonetheless, a number of obstacles still exist, such as the requirement for scalable synthesis techniques, improved long-term stability, and increased electrical conductivity. Significant gains in energy storage performance have been shown by combining MOF derivatives with composites, opening the door for more developments in this area. To fully realize MOFs’ promise for next-generation supercapacitor technologies, further research should concentrate on improving their electrochemical characteristics, investigating advanced hybrid materials, and optimizing their architectures. Resolving these issues will make it easier to use MOF-based materials in practice, which will assist in the development of systems for storing energy that are both high-performing and sustainable. Two significant Sustainable Development Goals (SDGs) of the UN are directly related to the phyto-synthesis of the AVL.A[Cu_2_OBi_2_O_3_ZrO_2_] nanocomposite for energy production and storage without the need for extra chemicals and reagents. First of all, it helps achieve Sustainable Development Goal 7 (SDG-7), which is to guarantee that everyone has access to modern, affordable, and sustainable energy. SDG 9 is further advanced by the AVL.A[Cu_2_OBi_2_O_3_ZrO_2_] nanocomposite’s enhanced stability, which encourages sustainable industry and resilient infrastructure. The SDGs that are pertinent to MOFs have a strong emphasis on a number of topics, such as innovation, infrastructure, responsible consumption, cheap and clean energy, climate action, and environmental impact. MOFs are essential to the development of cutting-edge energy storage and conversion technologies that support SDG 7 by expanding access to modern, clean, affordable, sustainable, and dependable energy sources. Innovation, constructing robust infrastructure, and encouraging sustainable industrialization are the main objectives of SDG 9. Building dependable, robust, sustainable, and high-quality infrastructure is one of the specific goals to boost economic development and significantly raise employment and GDP. Incorporating climate change measures into national plans, policies, planning, and adaptive capability is the focus of SDG 13. It includes carrying out the Paris Climate Agreement, which calls for halting the rise in average global temperatures while maintaining ongoing efforts to keep them from rising too far. The ultimate goal is to promote adaptation measures to address the effects of climate change and mitigation techniques to lower greenhouse gas emissions.

Biofuels, biomass, and bioenergy are essential for meeting the world’s energy needs and advancing the Sustainable Development Goals (SDGs) of the UN, while facilitating the shift to sustainable energy systems. The potential of biofuels to lower greenhouse gas emissions, improve energy security, and stimulate economic growth is highlighted in this article, which summarizes research on biofuels from first-to fourth-generation technology. Sustainable energy and climate resilience (SDGs 7, 12, and 13) and socioeconomic development and equity (SDGs 1, 8, and 15) are the two theme clusters identified by the analysis using the PRISMA framework, machine learning-based SDG mapping, and BERTopic modeling. Systemic innovation and process efficiency are highlighted in subjects such as supply chain optimization and bioeconomic development.

## Acknowledgments

A. Venkateswara Rao, the corresponding author expresses gratitude to the KLEF management, the 10.13039/501100001409Department of Science and Technology (DST), the Government of India, for providing the Department of Physics, KLEF, with the DST-FIST level-I (SR/FST/PS-I/2018/35), DST-PURSE (Grant No. SR/PURSE/2023/196), and DST-ANRF Grant No. EEQ/2023/001042 under the EEQ program for providing financial assistance, which the author recognizes.

## Author contributions

Literature review, article drafting, and graphical representation - P. Veerakumari. Conceptualization, supervision, project administration, and review and editing - A. Venkateswara Rao.; Literature collection and methodology support - K. Anjani Devi.; Data interpretation and article structuring - P. Venkata Rao.; Reference management and visualization - Shaik Raziya.; Validation and technical review - S. Shanmugan.; Proofreading and editing support - Arunkumar Jayakumar.; Graphical content preparation and formatting - Karthik Kannan.

## Declaration of interests

Authors declare that they have no known conflicting financial interests or personal ties that could have influenced any of the material presented in this study.
